# Book Review

**Published:** 1908-09

**Authors:** 


**Manual of Surgery.** By Alexis Thomson, and Alexander Miles. Edinburgh: Young J. Pentland. 1907. Vol. II. Second Edition. Pp. xiv., 816.—It is some three years since we reviewed the first edition of this book, the second volume of the Manual. The second edition has been enlarged by some eighty pages, and the illustrations increased by nearly fifty. The first addition we notice is the method of lumbar puncture with its various indications. Much is added with regard to the treatment of scoliosis by exercises. Nearly all the chapters have been enlarged, some of them renewed, but the number kept as before. The book has been brought well up to date, and is a most useful student's text-book.

**On Diseases of the Rectum and Anus.** Third Edition. Pp. xii., 588. **Cancer of the Rectum.** Fifth Edition. Pp. x., 255. By Harrison Cripps, F.R.C.S. London: J. and A. Churchill. 1907.—These may be taken together in review, and a table of

380 cases of cancer of the rectum is common to both books, a subject which occupies one-fourth of the larger volume. The special studies of the author, and the period they have covered, serve to perpetuate the authority of the works, and the many beautiful drawings by the author of microscopical sections enhance their value. It can scarcely be said that modern micro-photographic reproductions are so clear and instructive as these original drawings, which bring out special detail more impressively. From one of such experience in rectal surgery we may learn much from well-written monographs ; and concerning cancer of the rectum, he gives a mortality of 5 per cent. from radical operation, three out of every eight cases remained well over three years, and one patient was alive and well without recurrence for twenty-two years after excision. The text everywhere conveys the impression of being the product of years of hard work and matured experience, and combined with a pleasant literary style—a quality which seems to be rare in modern medical writings—it may be regarded as one of the most important literary achievements on regional surgery. We heartily commend the larger work to the notice of operating surgeons and practitioners.

**Surgery of the Rectum.** By FRED. C. WALLIS, M.D., F.R.C.S. Pp. vii., 168. London: Baillière, Tindall and Cox. 1907.—This is a very good little manual, fairly well illustrated, covering the general grounds of the surgery of the rectum, not intended for, nor adapted to, the requirements of operating surgeons. He emphasises the importance of abrasions and ulcerations of the proctodeum in the causation of abscesses and fistulæ, as well as of that troublesome affection, pruritus ani. There is a good diagram of such abscesses, the precursors of fistulæ, on page 23. Moreover, he regards the commoner strictures of the rectum as due to infection from ulceration, and not to constitutional disease. His statement that hemorrhoids are due to pathological changes in the veins requires some modification in the light of recent pathology. He gives preference to the operation of excision for piles, and states that the danger of stricture does not exist after it, provided proper after-treatment is carried out. This hardly accords with what we have seen resulting after the operation by well-known surgeons. He refers to the possibility of ulcerations of the rectum being the cause of recurrent synovitis of the larger joints. We are surprised to find that in the performance of colotomy he still sutures the peritoneum to the skin ; and are surprised also that, in common with some other surgeons, he finds that prolapse of the colon is not an uncommon occurrence. The former practice was ably contested by Greig Smith many years ago, and the latter result is rarely seen after operations done in this neighbourhood. It might be well if some surgeons seriously considered whether there are not means of operation in common use by which such an undesirable complication can be avoided. We have even known an examiner at the Embankment express surprise that a candidate was not aware that prolapse was a frequent complication. The book can be well recommended, it is interesting reading, it is not so deep nor so statistical as to be a soporific.

**Operations of General Practice.** By EDRED M. CORNER, M.C., M.B. (Cantab.), and H. IRVING PINCHES, M.B., B.C. (Cantab.). London: Henry Frowde and Hodder & Stroughton. 1907.—This is rather heavy as a manual, but is well printed, and clearly illustrated by line drawings and borrowed blocks. It is an attempt to meet a deficiency in professional education by the verbal description of classical and modern procedures in minor surgery. This is a worthy aim, but we think it is an impossible one, for even the details of minor surgery can only be properly appreciated by practical tuition in a hospital; and in the absence of such tuition we do not think a practitioner would be justified in amputating a breast or removing an exostosis of the femur from a perusal of the descriptions given, especially considering the warning given to the practitioner on page 276 not to scratch his unsterilised head or nose when assisting at an operation. There are many good points in the book, but the general practitioner will be wise to avoid attempting some of the operations described, unless he has had practical training and experience therein; and, amongst other things, we would advise him *not* to open the maxillary antrum through the canine fossa, dilate the communication with the nose by means of artery forceps passed into the nostril through the antrum, and make the patient dilate this opening night and morning with the terminal phalanx of his little finger (page 54).

**Surgical Emergencies.** By PERCY SARGENT, M.A., M.B., B.C. Pp. 256. London: Henry Frowde and Hodder & Stoughton. 1907.—This book, one of the Oxford Medical Series, is a handy little volume, but devoid of illustrations, and quite elementary in character, though a safe guide to many minor surgical emergencies. As stated in the preface, it reflects the teaching at St. Thomas's Hospital, and consequently we find the author giving preference to an abdominal incision by splitting the fibres of the right rectus muscle, though, speaking from experience, we do not think that this incision always has the advantages claimed for it. He speaks rather disparagingly of the treatment of compound fractures by antiseptics, and of excision of the gut for gangrenous hernia. His remarks on hemorrhage are judicious, but he does not mention the insertion of two fingers and the compression of the lingual against the cornu of the hyoid in hemorrhage from the tongue, nor the opening of a retropharyngeal abscess at the neck instead of through the mouth as advocated. However, the book is readable, generally accurate, and will prove of service to those for whom it is intended. There are a few slips, and a more careful editing and more copious index may be advantageous in any future edition. We note that he leaves out the diphthong — fasciae, haemorrhage, faecal, anaesthetic occur in the book. It is strange that in modern medical writings there should be various ways of spelling such words.

**The Diagnosis and Treatment of Intussusception.** By Charles P. B. Clubbe. Pp. viii., 92. Edinburgh and London: Young J. Pentland. 1907.—Mr. Clubbe has had an exceptionally large experience of intussusception, having had in thirteen years 144 cases under his care. He advocates a preliminary irrigation in all cases when the patient is anæsthetised ready for laparotomy, because even if the intussusception is of long standing, and a curative result is not to be expected, yet the irrigation reduces the invagination to a certain degree, and does so "in the best and gentlest possible way," and diminishes the need for manipulation of the intestine if laparotomy has to be performed. This method of treatment was carried out in 138 of the author's cases. In 14 of these irrigation alone was completely successful in effecting reduction; in the remaining 124 cases laparotomy was performed after irrigation. In three cases the abdominal exploration revealed that complete reduction had been effected by the irrigation. In one case only was irrigation possibly harmful, because it led to postponement of the operation for twenty-four hours, and the case ended fatally. In eight cases in which resection of bowel was performed one patient only survived, an infant eleven months of age. It is interesting to note that no less than 57 per cent. of the author's cases submitted to operation were examples of double intussusception. These double varieties included 42 cases of the cæco-ileocæcal type, in which the invagination begins at the caput cæci; 22 of the enteric-ileocæcal type, in which the invagination starts in the ileum near the ileocæcal valve; and 7 of the ileocolic-ileocæcal type, in which the intussuscepted ileum passes through the valve in the colon. We have read this short monograph with much pleasure and profit. It is full of sound practical hints and suggestive reasoning.

**Modern Methods of Diagnosis in Urinary Surgery.** By Edward Deanesly, M.D., B.Sc. Lond. Pp. iii., 97. London: H. K. Lewis. 1907.—The adjective "modern" in the title of this little book has special reference, we judge, to the description and advocacy of the use of cystoscopes and segregators for diagnosis in affections of the urinary organs. The description of the methods of using these instruments, and also that of the more ordinary methods of diagnosis, are succinct and methodical. In some places more or less original and suggestive opinions, the outcome of the writer's experience, find expression. Students and surgeons of limited experience, or deficient in scientific method, can be cordially recommended to appropriate the methodical system of investigation advocated by the author, and even those who are methodical and have "modern" experience will find food for reflection in the book. The chapter on the separate collection of urine from each kidney is particularly good, and in it the limitations of the segregator receive consideration, as well as its powers of achievement. Many valuable methods of diagnosis in urinary disease are not described; but the book is a short essay on diagnosis, not a treatise.

**The Johns Hopkins Hospital Reports : Studies in Urological Surgery**, Vol. XIII. ; **Studies on Hypertrophy and Cancer of the Prostrate**, Vol. XIV. Baltimore: The Johns Hopkins Press. 1906.—The thirteenth volume of these studies has been written by Drs. H. H. Young, A. A. Fowler, J. W. Churchman, J. T. Geraghty, L. C. Lehr, H. R. Stevens, S. H. Watts and F. H. Baetjer. The fourteenth volume is entirely the work of Dr. Hugh H. Young. Both volumes contain more than 600 closely-printed pages. Both are monuments of ability and industry, and demand the most careful consideration from all urologists. Dr. Young's results from perineal prostatectomy, with a total mortality of 7 in 238 cases and a series of 112 cases without a death, are the best yet obtained from any form of prostatectomy, and that fact alone shows how necessary it is to study his methods and researches. Among the subjects considered in these volumes are the seven-glass test, urethral diverticula, treatment of stricture of the urethra, operative treatment of vesical diverticula, chronic prostatitis, &c. We will not attempt criticism, but simply draw the attention of our readers to these important contributions to urological science.

**First Aid to the Injured.** By Dr. FRIEDRICH ESMARCH. Translated by H.R.H. Princess CHRISTIAN. Seventh Edition. Pp. xv., 138. London: Smith, Elder & Co. 1907.—This valuable little handbook continues to hold the field in face of many more pretentious rivals, and still reflects credit on the German savant who first ventured upon the unpopular experiment of teaching ambulance work to the multitude, and on the lady who, apart from translating the mere text, lent such influence as she possessed to fostering his schemes (not always without opposition, it must be confessed) in England. And now this book boasts of translations into twenty-seven other living languages, and has reached in all twenty-one editions since its first appearance in 1882. The author's name is a household word in English surgery, and we must confess that for precision, simplicity, and omission of what is unnecessary, even in England we unhesitatingly admit his pre-eminence. We like it the better, too, for the simple forceful language of the translation.

**"First Aid" to the Injured and Sick.** By F. J. WARWICK, M.B., and A. C. TUNSTALL, M.D. Fifth Edition. Pp. xiii., 252. Bristol: John Wright & Co. 1908.—When a book reaches its fifth edition there should be little room left for criticism, success being a fair test of value. The Order of St. John of Jerusalem, through its Ambulance Department, having now granted nearly a million certificates, is mainly responsible for the popularisation of first aid in this country, and with its fine organisation of medical and civilian helpers has largely overcome any tendency to the development of first-aiders into amateur practitioners of surgery, who might force their opinion into conflict with qualified medical men. This important position has been effected chiefly by the insistence of the Order that none of its certificate-holders shall dress an injury a second time, and also by arranging its teaching to be both simple and practical. We therefore consider that 68 pages of small print being devoted to anatomy and physiology to be out of place even in an *advanced* first aid manual, which this one is called. The chapter on the use of the triangular bandage for wounds (12 pages), and the same number for poisons, is at least a tax on the memory of the average person, and in the latter case an expansion of general rules for treatment would be much more useful. Again, practice with the roller bandage should be confined to the redressing of injuries by nurses, the skill required for its use being far more than a sound knowledge of first aid work with the triangular bandage demands. Without being captious, is the use of styptics advisable for first aid? and is "direct pressure by means of the fingers to the bleeding-point until the bleeding has quite stopped" desirable treatment for arterial hemorrhage? Again, we must dispute the wisdom of entertaining the risk of the use of the Esmarch's tube, or any other elastic or mechanical apparatus, for control of hemorrhage by a first-aider. We would point out that Schäfer does not suggest a pad under the patient's chest, or that his forehead should rest on his arm, in his method of artificial respiration; also the very out-of-date and dangerous method of short-circuiting an electric current by means of an iron rod. Drs. Warwick and Tunstall have made a brave attempt to comprise all that can be known in first aid within the pages of their book, but a smaller manual of general principles, couched in simple language which could easily be committed to memory, would be far more valuable to the man who is a practical and not a mere theoretical first-aider, and whose duty it is to confine himself within the limits of his art, and would not tend to fill him with knowledge which, however valuable, might confuse him in dealing with a serious emergency.

**Aids to Surgery.** By JOSEPH CUNNING, M.B., B.S. Second Edition. Pp. vi., 404. London: Baillière, Tindall & Cox. 1908.—This is a "cram" book, and as it comprises some 400

pages of small print, is somewhat voluminous as such. It might be of some service to men preparing for an examination, provided they have an intimate knowledge of standard works, so as to discard errors and evade false impressions. Apart from such use, we cannot recommend the book as a suitable one for students, although the author shows evidence in the text of considerable acquaintance with recent literature, and the work is more up to date than many of its class.

**Hernia: its Cause and Treatment.** By R. W. Murray, F.R.C.S. Pp. vi., 99. London: J. & A. Churchill. 1908.—In this small work the author advocates the "saccular theory" of causation of hernia as originally emphasised by Mr. Hamilton Russell. With this view, the desirability of getting rid of the sac is of the utmost importance, and operative methods are preferred in almost every case. The more complicated operations are rejected, and the procedure favoured is much the same as that advocated by the late Sir William Banks. There is not much that is new in the book, which has been mainly written to support the views to which reference has been made, and this results in a tendency to treat most cases in a stereotyped plan of operation, to the practical exclusion of treatment by means of trusses.

